# How Precise Is Predictive Coding for Things We Hear? Mismatch Negativity With Shepard Tones

**DOI:** 10.1111/ejn.70624

**Published:** 2026-07-07

**Authors:** Urte Roeber, Robert P. O'Shea

**Affiliations:** ^1^ Wilhelm Wundt Institute for Psychology, BioCog: Cognitive and Biological Psychology Leipzig University Leipzig Germany; ^2^ Discipline of Psychology, School of Psychology and Exercise Science Murdoch University Perth Australia

**Keywords:** audition, event‐related potentials (ERPs), mismatch negativity (MMN), predictive coding, Shepard tones

## Abstract

Predictive coding is a theory that each hierarchical level of the nervous system constructs a model from bottom‐up information (such as sensory inputs) and from top‐down information (such as expectations and reliability of past information) and then tests new inputs against the model. If the new inputs match the model, then no change in it is required. If not, then extra brain processing is required to update the model. We tested the precision of such a model in the auditory system by using Shepard tones arranged into a discrete Shepard scale—a series of notes, each comprising sine tones of different amplitudes and one octave apart, and with each tone separated from the next by one semitone, yielding a scale that ascends or descends forever. We unpredictably and occasionally replaced an expected tone in the scale by one that was two thirds of a semitone less, one third of a semitone less, one third of a semitone more, or two thirds of a semitone more. We measured the electrical activity of 20 participants' brains with 128 scalp electrodes (electroencephalography, EEG) while the tones were delivered to their ears. We found that event‐related potentials (ERPs) from 180–220 ms to these unpredictable tones were more negative the farther they were from the predicted note and more negative for tones that were less than the expected note. We conclude that the predictive model for the kind of regularity in a discrete Shepard scale has a sensitivity to tones less than one third of a semitone and is more sensitive to undershoots than to overshoots.

## Graphical abstract

MMN amplitudes to unpredictable tones in a discrete Shepard scale were more negative the farther the unpredictable tones were from the predicted note and more negative for tones that were less than the expected note. We conclude that the predictive model for a discrete Shepard scale has a sensitivity to tones less than one third of a semitone and is more sensitive to undershoots than to overshoots.

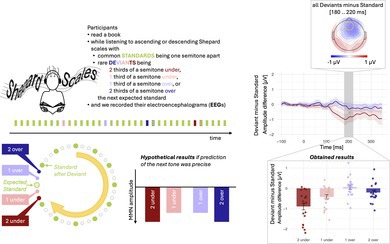

**Abbreviations**
ANOVA: analysis of variance
EEG: electroencephalography
ERP: event‐related potential
ICA: independent component analysis
MEG: magnetoencephalography
MMN: mismatch negativity
ROI: region of interest

## 1 Introduction

A popular technique for backing up information on one's computer is to begin by saving every file, and then, at regular intervals thereafter (such as daily) to check for changes and to save only the files that have changed. This is much more efficient than making a complete backup every time (Shannon 1948). Barlow (1961) proposed that nervous systems use a similar strategy to reduce redundancy of incoming sensory information but to respond to changes in that information. His proposal has grown into *predictive coding theory*, first for the mammalian visual system (Mumford 1992), then into the primate visual system (Rao and Ballard 1999), culminating in a general theory for all nervous systems based on the free‐energy principle (Friston 2010; Friston and Kiebel 2009). Key concepts of predictive coding theory are *predictive models* and *prediction errors*.

*Predictive models* are set up from bottom‐up information (such as sensory inputs) and from top‐down information (such as expectations and reliability of past information). These predictive models exist in various hierarchical parts of the nervous systems and are used to anticipate incoming information (see Millidge et al. 2022 for a recent review).

*Prediction errors* occur when the incoming information differs from the predictive model. When that happens, extra brain processing is required either to change the predictive model or to ignore the change because of strong top‐down information.

Predictive coding theory has grown to encompass numerous aspects of how human and animal brains function, of human experiences (e.g., consciousness, mind, speech, hallucinations, perception), and of different groups of humans (e.g., varying in age and in conditions—such as autism and psychopathology).

One of the hallmark signatures of prediction error has typically been extracted from electroencephalography (EEG) recordings to auditory stimuli: the *mismatch negativity* (MMN), a negative difference in event‐related potentials (ERPs) between rare, rule‐violating stimuli (*deviants*) and frequent, rule‐adherent stimuli (*standards*) (see Bartha‐Doering et al. 2015; Fitzgerald and Todd 2020 for recent reviews).

Although the concept of predictive coding theory is arguably appealing and there is a plethora of studies that provide evidence for its assumptions (but see Cao 2020; Feuerriegel 2024; May 2021 for critical evaluations), we have not come across studies that assess the sensitivity of a predictive model for things we hear. This is what we explore here.

If one listens to a series of musical notes in which each one is one semitone higher on the chromatic scale (e.g., C, C#, D, D#, E, E#, F, F#, A, and so on; each about 6% higher in pitch), then, at some level of the auditory system, a predictive model would be established encoding this regularity of the progression (i.e., upward) and interval (i.e., one semitone) between two subsequent tones. A new note that is unexpected, such as a repetition of a previous note (e.g., E followed by another E), or a reversal in the direction of the scale (e.g., E followed by D#) should require the model to be updated, involving extra brain processing (Garrido et al. 2009).

Tervaniemi et al. (1994) searched for evidence of this sort of extra processing using sine tones and Shepard tones (Shepard 1964) descending by one semitone steps between subsequent tones in a variant of the *oddball paradigm* used to measure MMN. Their participants listened to the tones while reading—that is, not paying attention to the tones. Occasionally, Tervaniemi et al. altered one note in a sequence (i.e., the downward progression by one semitone) by repeating a previous note or by increasing the pitch of a note by one semitone. They measured eight participants' brain activity from six electrodes on the scalp. When Tervaniemi et al. compared ERPs to the onset of the notes, they found greater MMNs around 130–220 ms to the unexpected, deviant tones than to the expected standard tones. The negativities were larger for the reversals than for the repetitions.^1^

To test the sensitivity of the predictive model (i.e., the encoding of the regular one semitone interval in the progression of a scale), we have conducted a study like Tervaniemi et al.'s, but using only discrete Shepard scales. This feature allowed us to keep the progression of the scale (Prince 2014; Shepard 1964) throughout an experimental block and therefore accumulate evidence for a predictive model of the regularity within the scale (i.e., one semitone steps in the same progression, ascending or descending). Crucially, and for the first time, we conducted a finer‐grained analysis of the sensitivity of the predictive model by preserving the scale's ascending or descending progression while varying the deviant notes to be either two thirds of a semitone less (i.e., close to a repetition, which was the smallest difference from expected that Tervaniemi et al. studied), one third of a semitone less (even smaller), one third of a semitone more, or two thirds of a semitone more than the expected tone. If the predictive model encodes interval information, these deviations should prompt prediction errors. We found that the sensitivity of the predictive model for musical scales is within one third of a semitone.

## 2 Method

### 2.1 Participants

Tervaniemi et al. (1994) did not give means, standard deviations or effect sizes for their results. We conducted an a priori power analysis (Faul et al. 2007) for a 2 × 2 repeated measures analysis of variance (ANOVA) (*α* = 0.05, *1‐ß* = 0.80) aiming at a moderate‐to‐large effect (*f* = 0.3) that resulted in a sample size of *N* = 17. To account for potential exclusions due to too many artifacts in the EEG data we collected data from 20 (10 female, 10 male) people who gave their informed consent to participate. Ages ranged from 19 to 63 (M = 28) years. Everyone had normal hearing and normal or corrected‐to‐normal vision. Nine participants were Psychology students of Murdoch University; they were required to participate in a chosen study for unit credit points. The remaining 11 were volunteers who had a chance to win a AU$20 gift voucher as reimbursement for participating. The ethics of the study were approved by Murdoch University's Human Research Ethics Committee (approval number 2016/112). We included the data of all 20 participants in our analyses.

### 2.2 Apparatus

Individual testing took place in a light‐ and sound‐attenuated cubicle within Murdoch University's Cognitive Neuroscience EEG Research Laboratory (https://sites.google.com/site/oshearobertp/research/laboratory‐facilities). Each participant sat in a comfortable chair and wore a pair of Sennheiser, HD25‐1 II, professional, on‐ear headphones through which the tones were delivered. Prior to putting on the headphones, the experimenter put a 128‐electrode, Hydro‐Cel Geodesic Sensor Net on the participant's head. The net was connected to the EGI system comprising a NetAmps 400 amplifier, a MacPro recording computer (3.7 GHz, Quad‐Core, Intel Xeon E5 with 10 MB L3 cache and Turbo Boost up to 3.9 GHz) running Netstation 5.2, and a Dell PC stimulating computer running especially written Octave (v4.0.0) scripts based on Psychtoolbox‐3 (Brainard 1997; Kleiner et al. 2007) under GNU Ubuntu (v16.04.4), Linux (v4.13.0).

### 2.3 Stimuli

There were 36 Shepard tones that differed by one third of a semitone. Each Shepard tone comprised 10 sinusoidal components one octave apart with amplitudes between 22 and 56 dB determined by a Gaussian around the central frequency. Frequencies of the components ranged between 9.73 and 9774 Hz. Shepard tones were 200 ms long with 10‐ms rise and fall times and were separated from the preceding tone by 250 ms. We provide the Shepard tones as wav‐files in the OSF repository's stimulation/wav folder (https://osf.io/gk6zs).

### 2.4 Procedure

**FIGURE 1:**
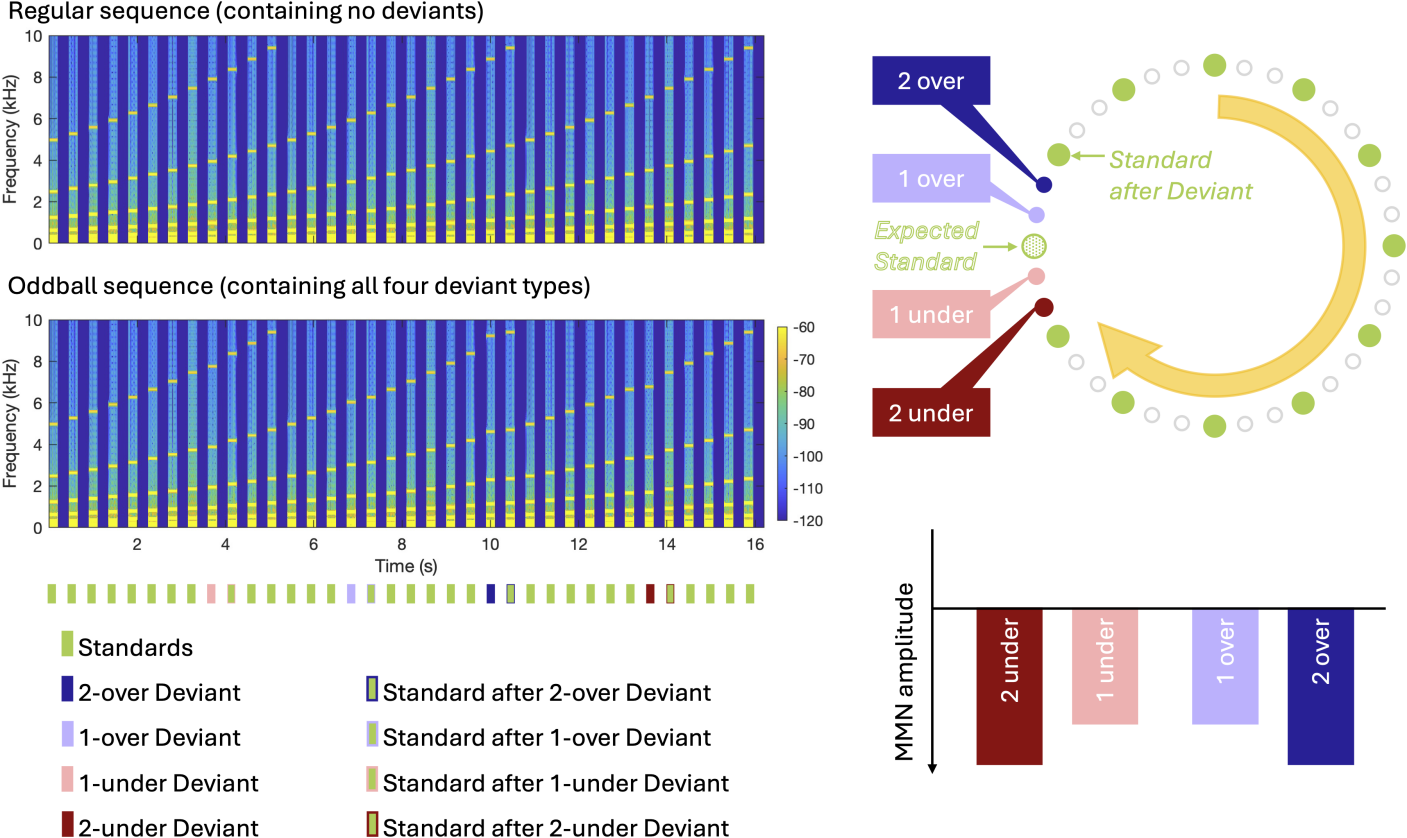
Design. The *right panel* shows time‐frequency‐plots of ascending stimulation sequences: the top plot depicts a purely regular sequence with no deviants (for comparison, not used in experiment); the bottom plot depicts the same sequence but with four standards replaced by one of the four different kinds of deviants (see colored rectangles underneath). The *left panel* shows a cartoon representation of the standard and four deviant conditions with the clock face representing the ever ascending or descending perception of the Shepard tones (top) and the expected pattern of MMN amplitudes for the deviants with different directions and magnitudes of deviance from the expected stimulus if prediction is precise.

Participants read a self‐chosen book during testing. There were nine blocks, each containing 586 standard tones (88%) and 80 deviant tones (12%) divided into 20 of each deviant type (3% each). Tones were arranged into sequences comprising at least three standard tones one semitone apart (either ascending or descending) followed by a deviant tone. Apart from that, length of the scale before a deviant and choice of deviant were randomized for sequence and each participant. There were four types of deviants: two thirds of a semitone less (i.e., one third of a semitone away from the previous standard), one third of a semitone less (i.e., two thirds of a semitone away from the previous standard), one third of a semitone more (i.e., four thirds of a semitone away from the previous standard), or two thirds of a semitone more (i.e., five thirds of a semitone away from the previous standard) than the expected note. After the deviant, a new sequence, going in the same direction, commenced at the next expected tone from the previous sequence (i.e., two semitones away from the previous standard and one, two, three, or five thirds of a semitone away from the deviant) so that the progression of the scale (ascending or descending) was the same for the block. We provide the oddball sequence (predshep_oddball.mp3) that is depicted in the spectrogram at the bottom of Figure 1 as a short example of the stimulation in the OSF repository's stimulation folder (https://osf.io/gk6zs).

Blocks took about 5 min. Participants could take a break between blocks, but few of them did. Half the participants began with an ascending scale in the first block; half began with a descending scale. Thereafter the progression of the scale alternated for each participant.

### 2.5 EEG Recording and Processing

For EEG recordings, we used EGI's NetAmps 400 amplifier connected to the 128‐channel geodesic electrode net, with the electrode at the standard Cz position serving as reference. Data acquisition was performed using NetStation 5.2 software at a sampling rate of 500 Hz with no online filtering applied. Before starting to record the data, we reduced impedances of all electrodes to below 5 kΩ.

We preprocessed the EEG data using EEGLAB (Delorme and Makeig 2004) with MatLab 2024a. We filtered the data with a low pass filter of 48 Hz (Kaiser window, transition bandwidth 4 Hz, filter order 454). In preparation for the following analyses, we filtered the data additionally with a high‐pass filter of 0.1 Hz (Kaiser window, transition bandwidth 0.2 Hz, filter order 9056) and extracted epochs from 100 ms before to 350 ms after tone onset. We used a modified version of the *findNoisyChannels()* function from the *prepPipeline* plugin (Bigdely‐Shamlo et al. 2015) implementation in EEGLAB to detect automatically channels with excessive noise (*z* score estimate of noise‐to‐signal ratio > 5, which refers to ratio of power above 50 Hz to total signal power), extreme amplitudes (robust channel deviation *z* score > 5), or too low correlation with other channels' data (percentage of epochs with a correlation < 0.4 was above 1%) with the exception of electrodes surrounding the eyes (E1, E8, E14, E17, E21, E25, E32, E126, E127)—because they were required for ocular artifact component detection, on the mastoids (E57, E100), or at the fringe of the net (E38, E43, E48, E49, E56, E63, E68, E73, E81, E88, E94, E99, E107, E113, E119, E120, E121, E125)—because they cannot be interpolated. Identified bad channels were excluded from subsequent preprocessing and later interpolated using spherical splines to preserve topographical information. We also removed epochs that contained amplitude changes of more than 600 μV, which are most likely due to non‐stereotypical artifacts such as swallowing, but retained epochs containing stereotypical artifacts such as eye movements and blinks to be removed via independent component analysis (ICA) artifact correction.

For ICA we also applied a low pass filter of 48 Hz (Kaiser window, transition bandwidth 4 Hz, filter order 454) to the raw data, but this time followed by a high‐pass filter of 1 Hz (Kaiser window, transition bandwidth 0.5 Hz, filter order 3624). We extracted the same epochs and removed the same noisy channels and epochs with non‐stereotypical artifacts as identified before. We confirmed that the rank of each dataset agreed with the number of its retained channels (between 119 and 128). We ran an ICA with the adaptive mixture‐independent component analysis (AMICA) algorithm (Palmer et al. 2011). We copied the ICA unmixing matrix to the previously prepared (0.1‐Hz high‐pass filtered) data that we used for subsequent analyses. Using the *dipfit 5.6* plugin a single equivalent current dipole was fitted for each independent component, and subsequently symmetrically constrained dipoles were identified using the *fitTwoDipoles v1.00* plugin (Piazza et al. 2016). We did this to improve the classification of the independent components employing the *ICLabel* plugin (Pion‐Tonachini et al. 2019). With this we removed from the data any component that had a probability to be of ocular origin of more than 70% if its probability of originating from the brain was less than 5%. We removed between 2 and 6 (mean = 3.5) components per participant.

We baseline corrected all epochs with a −100 to 0 ms baseline, removed any epoch that exhibited amplitude changes larger than 150 μV, interpolated all channels that were previously excluded, and rereferenced the data to the average of all channels except those surrounding the eyes.

Finally, we separated epochs by stimulus type: standards (excluding standards after a deviant), 2‐under deviants, 1‐under deviants, 1‐over deviants, and 2‐over deviants. For each stimulus type, we calculated individual ERPs, which we then averaged across participants. Each individual ERP contained at least 110 trials. Individual ERPs contained a mean number of 3361 ± 391 (SD) standard, 160 ± 19 2‐under deviant, 158 ± 20 1‐under deviant, 159 ± 18 1‐over deviant, and 159 ± 21 2‐under deviant epochs.

For further processing, visualization, and statistical analyses of the data, we used R (Version 4.3.2; R Core Team, 2024) via RStudio. We used the tidyverse package suite (Wickham et al. 2019) for data manipulation and visualization, including ggplot2 and gghalves for plotting. We performed frequentist repeated measures ANOVAs using the afex (Singmann et al. 2025) and ez (Lawrence 2016) packages in R and Bayesian ANOVAs in JASP (with default scaling factor *r* = 0.5 for fixed effects and *r* = 1 for the participant random effect).

To assess MMN elicitation and its modification by direction and magnitude of deviation, we defined the electrode at the standard Fz position (E11) and the seven electrodes surrounding it (E4, E5, E10, E12, E16, E18, E19) as our region of interest (ROI). The data we show and analyze were averaged across those seven electrodes. We chose this ROI to allow easy comparison with Tervaniemi et al.'s (1994) results, who evaluated their MMNs at Fz.

To determine the MMN time window, we subtracted the grand‐average ERP to standard tones from the grand‐average ERP to all deviant tones irrespective of their direction or magnitude of deviation. From this difference wave we took the latency of the largest negative deviation between 100 and 250 ms after tone onset and defined the MMN time window from 20 ms before to 20 ms after this peak, which was at 200 ms after tone onset. Hence, the time window we used for MMN analysis is from 180 to 220 ms after tone onset.

For statistical analysis, we subtracted the grand‐average ERP to standard tones from the grand‐average ERP of each deviant type. For these differences we averaged the difference amplitudes across the MMN time window to yield the MMN amplitude for each deviant type. To evaluate whether MMN amplitudes were significantly less than zero, we conducted frequentist as well as Bayesian one‐sample *t*‐tests.

To answer our research question we conducted a 2 × 2 repeated measures ANOVA with the within‐participants factors of direction (under, over) and magnitude (small, large) and its Bayesian equivalent. With the Bayesian analysis we examined the same 2 × 2 design with within‐participants factors of direction (under, over) and magnitude (small, large), treating participant as a random factor. The Bayesian approach compared multiple nested models and the null model containing only the subject random effect against the best model. It provides Bayes Factors for each main effect and interaction. Models were evaluated using default JZS priors. We interpret Bayes Factors according to conventional evidence thresholds (Lee and Wagenmakers 2014, p. 105; see also Jeffreys 1961, p. Appendix B).

## 3 Results

### 3.1 ERPs

**FIGURE 2:**
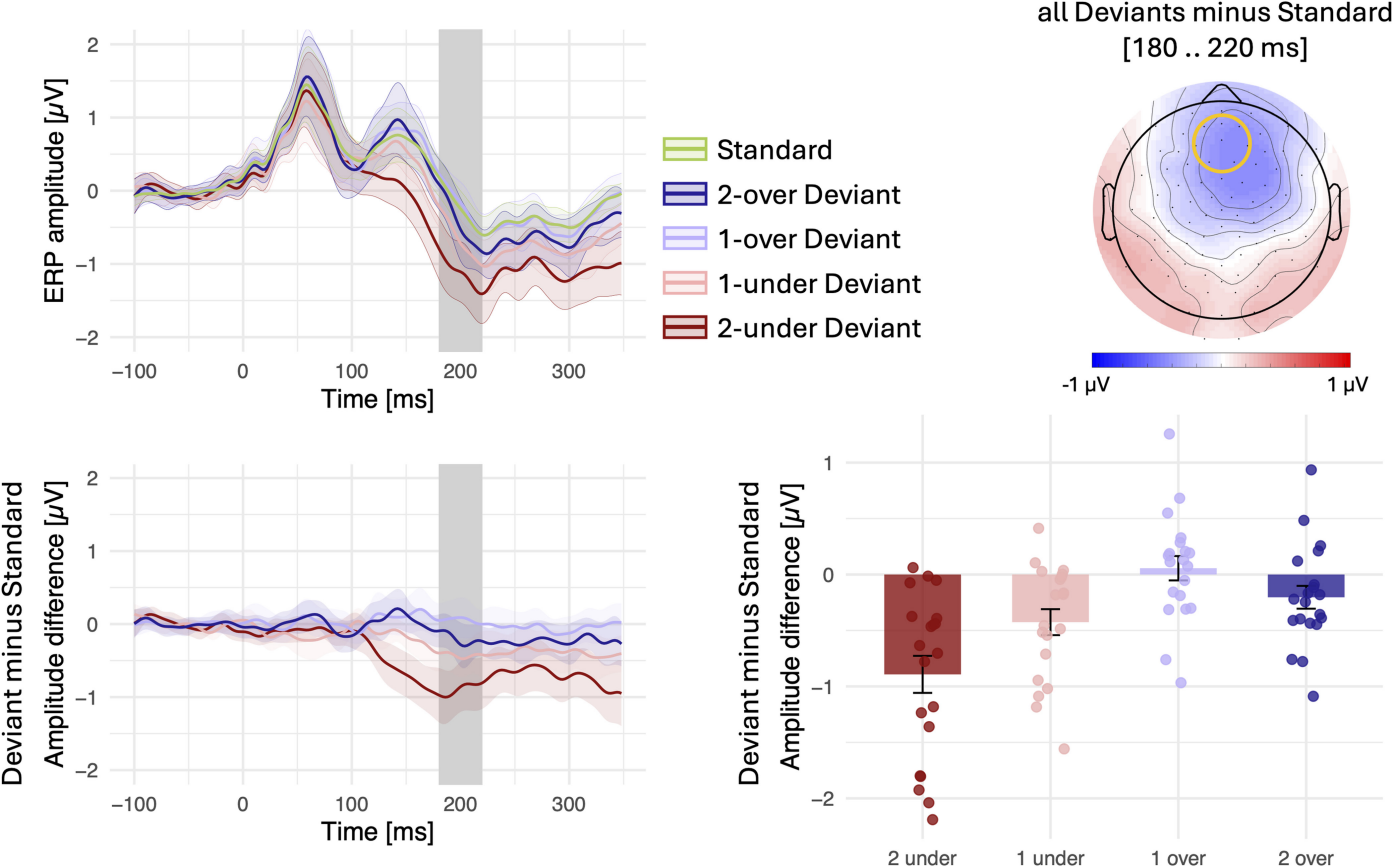
ERP results. *Top left*: ERPs to standards and all four deviants at the ROI around Fz. The same desaturated colored shading around each the five ERP traces show ±1 standard error of the mean. The grey shaded rectangle shows the MMN time window (180–220 ms after stimulus onset). *Top right*: Voltage map of the deviant (irrespective of type) minus standard difference in the MMN time window. The yellow circle marks the ROI around Fz. *Bottom left*: Four deviant‐minus‐standard difference waves at the ROI around Fz. *Bottom right*: Bar graphs of the mean amplitudes in the MMN time window. Error bars show standard errors. Dots show single participant data.

We show the ERPs at the Fz ROI for the standards and four different types of deviants in the top left panel of Figure 2. All ERPs show a positive deflection at about 50 ms (P1) after tone onset (latency 0 ms), a negative deflection at about 100 ms (N1), and another positive deflection at about 140 ms (P2). It is here when the ERPs start to differ from each other, most obviously the 2‐under deviant from the others. This can be seen more clearly in the standard minus deviant difference waves depicted in the bottom left panel in Figure 2. We determined the MMN time window by subtracting the grand‐average ERP to standards from the grand‐average ERP to all deviants irrespective of their type (not shown in the figure) and finding the peak negative difference between 100 and 250 ms at the Fz ROI. This peak was at 200 ms after tone onset, we set the MMN time window to ±20 ms around this peak (i.e., from 180 to 220 ms) as depicted in the grey shaded areas. The topographic map in the top right panel shows the difference in voltage between all deviants and standards in this time window. The voltage distribution shows a frontally distributed negativity which is typical for auditory MMN. However, the bar graphs of the mean difference amplitude for the different types of deviants in the right bottom panel of Figure 2, show stark differences in amplitude depending on direction and magnitude of deviance from the expected standard. That is, there is a clear MMN for the 2‐under deviants and a smaller MMN for the 1‐under deviants. But there are essentially no MMN for the 1‐over and very little for the 2‐over deviants (see Table 1 for the descriptive and inferential statistics).

**TABLE 1:** Descriptive and inferential statistics of the MMN amplitudes (Deviant minus Standard) within the 180‐ to 220‐ms MMN time window at the FZ ROI. We give the *t* statistics and Bayes factor (*BF*_10_: in favor of H1 [mean < 0] vs. H0 [mean ≥ 0]) for one‐sided tests against zero.

	Mean amplitude (μV)	Standard deviation	Cohen's *d*	*t*(19)	*p*	*BF*_−0_
2 under	−0.97	0.74	−1.30	−5.81	< 0.001	3371
1 under	−0.49	0.50	−0.97	−4.36	< 0.001	188.2
1 over	0.03	0.48	0.06	0.27	0.60	0.20
2 over	−0.21	0.48	−0.44	−1.95	0.03	2.14

*Note:* Evidence interpretation (Lee and Wagenmakers 2014, p. 105): 1/10 ≤ *BF* < 1/3: Moderate evidence for H0, 1 ≤ *BF* < 3: Anecdotal evidence for H1, 3 ≤ *BF* < 10: Moderate evidence for H1, 10 ≤ *BF* < 30: Strong evidence for H1, 30 ≤ *BF* < 100: Very strong evidence for H1, BF ≥ 100: Extreme evidence for H1.

The repeated measures ANOVA yielded a significant main effect of direction (under vs. over expectation), *F*(1,19) = 25.53, *p* < 0.001, generalized *η* ^*2*^ = 0.25, with deviants with smaller intervals to the preceding standard than expected showing larger amplitudes than deviants with larger intervals. It also yielded a significant main effect of magnitude of deviance (small = 1 third or large = 2 thirds of a semitone away from the expected standard), *F*(1,19) = 13.00, *p* = 0.002, generalized *η* ^*2*^ = 0.10, with deviants with larger intervals to the expected standard showing larger amplitudes than deviants with smaller intervals to the expected standard. There was no significant interaction of direction and magnitude, *F*(1,19) = 0.96, *p* = 0.339, generalized *η* ^*2*^ = 0.01. We used Bayesian repeated‐measures ANOVA to determine which factors best explained our results while accounting for individual differences between participants. As with the frequentist ANOVA, the analysis preferred the additive model including both main effects, direction and magnitude (*P(M|data*) = 0.569, *BF*_10_ = 1.0). Adding the direction by magnitude interaction term reduced model support (*P(M|data) = 0.339*, *BF*_10_ *= 0.596*), indicating little evidence for an interaction between magnitude and direction. Models including only *direction* (*P(M|data) = 0.090, BF*_10_ *= 0.159*) or only *magnitude* (*P(M|data) = 0.002, BF*_10_ *= 0.004*) were poorly supported. The null model received virtually no support (*P(M|data) = 3.712 × 10* ^*−4*^ *, BF*_10_ *= 6.529 × 10* ^*−4*^). When we examined each factor individually, direction alone showed extreme evidence of an effect (*BF*_*incl*_ = 274.1), whereas magnitude alone showed only moderate evidence (*BF*_*incl*_ = 6.689), and the direction by magnitude interaction showed only anecdotal evidence (*BF*_*incl*_ = 2.049).

## 4 Discussion

We set out to examine the sensitivity of a predictive model to changes in auditory input from standard Shepard tone scales with tones differing frequently by one semitone to tones that varied in magnitude of deviance, rare deviants, by two thirds of a semitone less, one third of a semitone less, one third of a semitone more, and two thirds of a semitone more than predicted by the model. If the predictive model accurately predicted the incoming stimulus—with the tone being a semitone higher (or lower) than the last—we anticipated that deviants of two thirds of a semitone more or less than predicted would yield the largest MMNs and that deviants of one third of a semitone more or less than predicted would yield smaller, but still significant MMNs. We found that deviants with smaller intervals than predicted (undershoots) behaved as we expected, but that deviants with larger intervals than those predicted (overshoots) did not. There was a weak MMN for overshoot deviants two thirds above but none for one third above the predicted semitone. Why?

**FIGURE 3:**
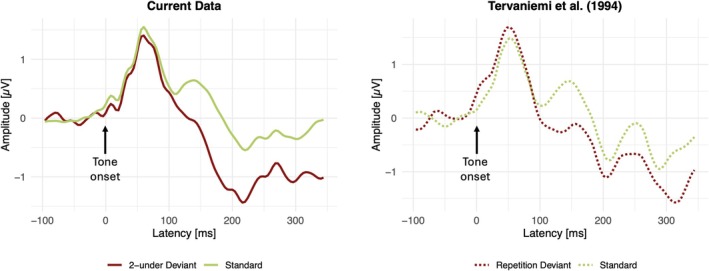
Comparison of the ERPs from the current study (left panel) with the ERPs from Tervaniemi et al.'s Shepard tone condition (right panel). Standards in both studies are Shepard tones spaced one semitone apart (we used both ascending and descending; Tervaniemi et al. used only descending scales). We show the 2‐under deviant (one third of a semitone from the preceding tone) of our study is the one most alike to the repetition deviant of Tervaniemi et al.'s study.

It is possible that undershoot deviants present more of a challenge to the model than overshoot deviants, requiring extra processing to remedy the prediction error. Indeed, deviants with two thirds of a semitone less than the predicted were the most salient, sounding a lot like a repetition of the previous standard. Tervaniemi et al. (1994) found that an unexpected repetition within a Shepard tone scale yields a convincing MMN. In Figure 3, we compare our result with theirs—they are remarkably similar. This provides a conceptual replication of Tervaniemi et al.'s results from a much smaller sample and comfortingly suggests that our paradigm worked.

However, we were not successful in finding any MMN study in which subsequent standards differed from each other by both a fixed interval and a consistent ascending or descending progression. In our paradigm, each subsequent tone in an ascending (or descending) scale was consistently 6% higher (or lower) in frequency than the preceding tone. The deviants, in contrast, differed only in interval size while preserving the same progression of frequency change. Hence, for now we can only speculate about the differences in sensitivity of the predictive model for undershoots and overshoots.

One of the longstanding issues in MMN research is that MMN is due to differences in adaptation between standard and deviant tones (see e.g., May 2021; Willmore and King 2023 for recent accounts). However, carefully designed control conditions (Ruhnau et al. 2012; Widmann et al. 2026; Wolff and Schröger 2001) provide evidence for genuine MMN responses that cannot be explained by adaptation alone and are instead consistent with a prediction‐error account of MMN.

We did not explicitly control for adaptation in the present design. If adaptation nevertheless contributed to our findings, we would expect one of the following three patterns. First, assuming adaptation is strongest for the immediately preceding tone, MMNs should increase with increasing difference in pitch between the deviant and the preceding standard, that is, for the overshoots. Second, assuming adaptation operates at the level of semitone intervals, MMNs should be largest for the 2‐under and 2‐over deviants, which differ most strongly from the expected standard. Third, assuming adaptation affects the 12 Shepard tones constituting the standard Shepard‐tone scale, MMNs should be of similar size across all deviant types. The latter account appears unlikely, given that each tone of the standard scale occurred with a probability of only 7.3% within a block. More importantly, our observed pattern of results is inconsistent with any of these three adaptation mechanisms. Hence, adaptation does not help to explain the mystery that undershoots yield larger MMNs than overshoots.

According to predictive coding theory, prediction error scales with the confidence (precision weighting) assigned to the predictive model (Clark 2016; Millidge et al. 2022; Yeark et al. 2021): The less confidence the brain has in its model or the more volatile the environment is perceived to be the more muted is the prediction error response, for example, by MMN amplitude (SanMiguel et al. 2021). Again, it remains unclear why the confidence concerning tonal intervals should be greater for undershoots than for overshoots. One possibility is suggested by evidence that, when tuning instruments, intervals slightly larger than the exact chromatic ratio may be perceived as more pleasant (Hergert 2024). If so, overshoots may be less readily perceived as deviations than undershoots because they fall within a more acceptable range of variability. However, Siegel and Siegel (1977) reported that even “musicians can't tell flat from sharp.” More importantly, differential precision weighting appears unlikely in our paradigm because the predictive model cannot determine whether a forthcoming deviant will be an undershoot or an overshoot until the deviant actually occurs. At present, therefore, we lack a compelling predictive‐coding account of the asymmetry observed between undershoots and overshoots.

One avenue for future research concerns the relationship between our findings and the broader literature on predictive processing in music. Previous work has shown that listeners develop predictive models for conventional music (e.g., Koelsch et al. 2019; Vuust et al. 2022). These models show that various ERP components appear when aspects of music occasionally and unexpectedly differ from prior music in aspects such as tempo, melody, and harmony. These include various early ERPs such as the MMN and later ERPs (e.g., Koelsch et al. 2019). Other research has used magnetoencephalography (MEG) allowing brain regions active for musical prediction errors to be identified, for memory of tunes(e.g., Bonetti et al. 2025; Greco et al. 2024), and for differences among individuals with various levels of training in music (e.g., Bonetti et al. 2024).

Here, we merely used a simple form of music as a tool to investigate the extent to which predictive models encode fine‐grained auditory structure. We did not ask the participants about their prior training in music. If we had, it is doubtful we would have found any significant correlation with only 20 participants. This could be rectified in future research. A larger sample size might also help to determine the stability and generalisability of the observed effect. We are running a study similar to this right now—its preliminary analyses confirm the pattern of the current results, apparently with no modulation by musicality.

Despite the mysterious asymmetry between undershoots and overshoots, there was a weak MMN for the largest overshoot, consistent with the predictive model modulated by poor precision of prediction. If so, then our prediction shown in the cartoon of Figure 1 was not too bad. MMNs can indeed be produced by deviants of one third of a semitone.

To strengthen the predictive‐coding claim, future research could include additional analyses/controls including equiprobable/many‐standards control or trial‐wise modelling of expectancy. Although our thoughts are speculative, we are planning to explore MMNs to standard sine‐tone pairs showing a one semitone increase with unexpected deviant pairs in which the second tone is an undershoot or an overshoot. We hope, dear reader, you will be as interested in what we find as we are.

## Author Contributions

**Urte Roeber:** conceptualization, methodology, software, validation, formal analysis, investigation, resources, data curation, writing – original draft, writing – review and editing, visualization, supervision, project administration. **Robert P. O'Shea:** conceptualization, writing – original draft, writing – review and editing, supervision.

## Funding

This work was supported by Murdoch University.

## Conflicts of Interest

The authors declare no conflicts of interest.

## Data Availability

The aggregated data including the code used for data analysis are provided in the OSF repository (https://osf.io/gk6zs) in JASP format (and the corresponding html output). The raw data are available from the corresponding author upon request.

## Endnotes

^1^
We have been able to find only one other study the authors of which used Shepard tones to look for the MMN, although we stand to be corrected on this. Tsogli et al. (2019) used Shepard tones but they did not manipulate their frequencies for the MMN; rather they manipulated the tones' transitional probabilities or ear to which the tones were delivered to create deviants.

## References

[ejn70624-bib-0001] Barlow, H. B. 1961. “Possible Principles Underlying the Transformation of Sensory Messages.” Sensory Communication 1, no. 1: 217–233.

[ejn70624-bib-0002] Bartha‐Doering, L. , D. Deuster , V. Giordano , A. am Zehnhoff‐Dinnesen , and C. Dobel . 2015. “A Systematic Review of the Mismatch Negativity as an Index for Auditory Sensory Memory: From Basic Research to Clinical and Developmental Perspectives.” Psychophysiology 52, no. 9: 1115–1130. 10.1111/psyp.12459. 26096130

[ejn70624-bib-0003] Bigdely‐Shamlo, N. , T. Mullen , C. Kothe , K.‐M. Su , and K. A. Robbins . 2015. “The PREP Pipeline: Standardized Preprocessing for Large‐Scale EEG Analysis.” Frontiers in Neuroinformatics 9, no. June: 16. 10.3389/fninf.2015.00016. 26150785 PMC4471356

[ejn70624-bib-0004] Bonetti, L. , G. Fernández‐Rubio , M. H. Andersen , et al. 2025. “BROAD‐NESS Uncovers Dual‐Stream Mechanisms Underlying Predictive Coding in Auditory Memory Networks.” Advanced Science 12, no. 44: e07878. 10.1002/advs.202507878. 41020476 PMC12667469

[ejn70624-bib-0005] Bonetti, L. , G. Fernández‐Rubio , F. Carlomagno , et al. 2024. “Spatiotemporal Brain Hierarchies of Auditory Memory Recognition and Predictive Coding.” Nature Communications 15, no. 1: 4313. 10.1038/s41467-024-48302-4. PMC11109219 38773109

[ejn70624-bib-0006] Brainard, D. H. 1997. “The Psychophysics Toolbox.” Spatial Vision 10, no. 4: 433–436. 10.1163/156856897X00357. 9176952

[ejn70624-bib-0007] Cao, R. 2020. “New Labels for Old Ideas: Predictive Processing and the Interpretation of Neural Signals.” Review of Philosophy and Psychology 11, no. 3: 517–546. 10.1007/s13164-020-00481-x.

[ejn70624-bib-0008] Clark, A. 2016. Surfing Uncertainty: Prediction, Action, and the Embodied Mind. Oxford University Press. 10.1093/acprof:oso/9780190217013.001.0001.

[ejn70624-bib-0009] Delorme, A. , and S. Makeig . 2004. “EEGLAB: An Open Source Toolbox for Analysis of Single‐Trial EEG Dynamics Including Independent Component Analysis.” Journal of Neuroscience Methods 134, no. 1: 9–21. 10.1016/j.jneumeth.2003.10.009. 15102499

[ejn70624-bib-0010] Faul, F. , E. Erdfelder , A.‐G. Lang , and A. Buchner . 2007. “G*Power 3: A Flexible Statistical Power Analysis Program for the Social, Behavioral, and Biomedical Sciences.” Behavior Research Methods 39, no. 2: 175–191. 10.3758/BF03193146. 17695343

[ejn70624-bib-0011] Feuerriegel, D. 2024. “Adaptation in the Visual System: Networked Fatigue or Suppressed Prediction Error Signalling?” Cortex 177: 302–320. 10.1016/j.cortex.2024.06.003. 38905873

[ejn70624-bib-0012] Fitzgerald, K. , and J. Todd . 2020. “Making Sense of Mismatch Negativity.” Frontiers in Psychiatry 11, no. June: 468. 10.3389/fpsyt.2020.00468. 32595529 PMC7300203

[ejn70624-bib-0013] Friston, K. 2010. “Is the Free‐Energy Principle Neurocentric?” Nature Reviews Neuroscience 11, no. 8: 605. 10.1038/nrn2787-c2. 20631713

[ejn70624-bib-0014] Friston, K. , and S. Kiebel . 2009. “Predictive Coding Under the Free‐Energy Principle.” Philosophical Transactions of the Royal Society of London. Series B, Biological Sciences 364, no. 1521: 1211–1221. 10.1098/rstb.2008.0300. 19528002 PMC2666703

[ejn70624-bib-0015] Garrido, M. I. , J. M. Kilner , K. E. Stephan , and K. J. Friston . 2009. “The Mismatch Negativity: A Review of Underlying Mechanisms.” Clinical Neurophysiology 120, no. 3: 453–463. 10.1016/j.clinph.2008.11.029. 19181570 PMC2671031

[ejn70624-bib-0016] Greco, A. , J. Moser , H. Preissl , and M. Siegel . 2024. “Predictive Learning Shapes the Representational Geometry of the Human Brain.” Nature Communications 15, no. 1: 9670. 10.1038/s41467-024-54032-4. PMC11549346 39516221

[ejn70624-bib-0017] Hergert, F. 2024. “Targeted Detuning Aiming for Sensory Pleasantness—A Case Study of Pipe Organs and Accordions.” Acta Acustica 8: 33. 10.1051/aacus/2024020.

[ejn70624-bib-0018] Jeffreys, S. H. 1961. The Theory of Probability. Third ed. Oxford University Press.

[ejn70624-bib-0019] Kleiner, M. , D. Brainard , D. Pelli , A. Ingling , R. Murray , and C. Broussard . 2007. “What's New in Psychtoolbox‐3.” Perception 36 (ECVP Abstract Supplement): 14.

[ejn70624-bib-0020] Koelsch, S. , P. Vuust , and K. Friston . 2019. “Predictive Processes and the Peculiar Case of Music.” Trends in Cognitive Sciences 23, no. 1: 63–77. 10.1016/j.tics.2018.10.006. 30471869

[ejn70624-bib-0021] Lawrence, M. A. 2016. ez: Easy Analysis and Visualization of Factorial Experiments (Version 4.4‐0) [Computer Software]. Comprehensive R Archive Network (CRAN). https://cran.r‐project.org/web/packages/ez/index.html.

[ejn70624-bib-0022] Lee, M. D. , and E.‐J. Wagenmakers . 2014. Bayesian Cognitive Modeling: A Practical Course. Cambridge University Press. 10.1017/CBO9781139087759.

[ejn70624-bib-0023] May, P. J. C. 2021. “The Adaptation Model Offers a Challenge for the Predictive Coding Account of Mismatch Negativity.” Frontiers in Human Neuroscience 15, no. November: 721574. https://www.frontiersin.org/articles/. 10.3389/fnhum.2021.721574. 34867238 PMC8640521

[ejn70624-bib-0024] Millidge, B. , A. Seth , and C. L. Buckley . 2022. “Predictive Coding: A Theoretical and Experimental Review.” (arXiv:2107.12979). arXiv. 10.48550/arXiv.2107.12979.

[ejn70624-bib-0025] Mumford, D. 1992. “On the Computational Architecture of the Neocortex.” Biological Cybernetics 66, no. 3: 241–251. 10.1007/BF00198477. 1540675

[ejn70624-bib-0026] Palmer, J. , K. Kreutz‐Delgado , and S. Makeig . 2011. AMICA: An Adaptive Mixture of Independent Component Analyzers With Shared Components. Swartz Center for Computational Neuroscience (SCCN). https://www.semanticscholar.org/paper/AMICA‐%3A‐An‐Adaptive‐Mixture‐of‐Independent‐with‐Palmer‐Kreutz‐Delgado/5774e96ad450c228400dc311f16caf1f20967c10.

[ejn70624-bib-0027] Piazza, C. , M. Miyakoshi , Z. Akalin‐Acar , et al. 2016. “An Automated Function for Identifying EEG Independent Components Representing Bilateral Source Activity.” In XIV Mediterranean Conference on Medical and Biological Engineering and Computing 2016, edited by E. Kyriacou , S. Christofides , and C. S. Pattichis , 105–109. Springer International Publishing. 10.1007/978-3-319-32703-7_22.

[ejn70624-bib-0028] Pion‐Tonachini, L. , K. Kreutz‐Delgado , and S. Makeig . 2019. “ICLabel: An Automated Electroencephalographic Independent Component Classifier, Dataset, and Website.” NeuroImage 198: 181–197. 10.1016/j.neuroimage.2019.05.026. 31103785 PMC6592775

[ejn70624-bib-0029] Prince, J. 2014. “Circular Tones.” In Music in the Social and Behavioral Sciences: An Encyclopedia (Vol. 2), edited by W. F. Thompson , 178–180. SAGE Publications, Inc. 10.4135/9781452283012.n63.

[ejn70624-bib-0030] Rao, R. P. N. , and D. H. Ballard . 1999. “Predictive Coding in the Visual Cortex: A Functional Interpretation of Some Extra‐Classical Receptive‐Field Effects.” Nature Neuroscience 2, no. 1: 79–87. 10.1038/4580. 10195184

[ejn70624-bib-0045] R Core Team . 2024. R: A Language and Environment for Statistical Computing. R Foundation for Statistical Computing. https://www.R‐project.org/.

[ejn70624-bib-0031] Ruhnau, P. , B. Herrmann , and E. Schröger . 2012. “Finding the Right Control: The Mismatch Negativity Under Investigation.” Clinical Neurophysiology 123, no. 3: 507–512. 10.1016/j.clinph.2011.07.035. 21839676

[ejn70624-bib-0032] SanMiguel, I. , J. Costa‐Faidella , Z. R. Lugo , E. Vilella , and C. Escera . 2021. “Standard Tone Stability as a Manipulation of Precision in the Oddball Paradigm: Modulation of Prediction Error Responses to Fixed‐Probability Deviants.” Frontiers in Human Neuroscience 15, no. September: 734200. 10.3389/fnhum.2021.734200. 34650417 PMC8505747

[ejn70624-bib-0033] Shannon, C. E. 1948. “A Mathematical Theory of Communication.” Bell System Technical Journal 27, no. 3: 379–423. 10.1002/j.1538-7305.1948.tb01338.x.

[ejn70624-bib-0034] Shepard, R. N. 1964. “Circularity in Judgments of Relative Pitch.” Journal of the Acoustical Society of America 36, no. 12: 2346–2353. 10.1121/1.1919362.

[ejn70624-bib-0035] Siegel, J. A. , and W. Siegel . 1977. “Categorical Perception of Tonal Intervais: Musicians Can't Tell Sharp From Flat.” Perception & Psychophysics 21, no. 5: 399–407. 10.3758/BF03199493.

[ejn70624-bib-0036] Singmann, H. , B. Bolker , J. Westfall , et al. 2025. afex: Analysis of Factorial Experiments (Version 1.5‐0) [Computer Software]. CRAN. https://cran.r‐project.org/web/packages/afex/index.html.

[ejn70624-bib-0037] Tervaniemi, M. , S. Maury , and R. Näätänen . 1994. “Neural Representations of Abstract Stimulus Features in the Human Brain as Reflected by the Mismatch Negativity.” Neuroreport 5, no. 7: 844–846. 8018861 10.1097/00001756-199403000-00027

[ejn70624-bib-0044] Tsogli, V. , S. Jentschke , T. Daikoku , and S. Koelsch . 2019. “When the Statistical MMN Meets the Physical MMN.” Scientific Reports 9, no. 1: 5563. 10.1038/s41598-019-42066-4. 30944387 PMC6447621

[ejn70624-bib-0038] Vuust, P. , O. A. Heggli , K. J. Friston , and M. L. Kringelbach . 2022. “Music in the Brain.” Nature Reviews Neuroscience 23, no. 5: 287–305. 10.1038/s41583-022-00578-5. 35352057

[ejn70624-bib-0039] Wickham, H. , M. Averick , J. Bryan , et al. 2019. “Welcome to the Tidyverse.” Journal of Open Source Software 4, no. 43: 1686. 10.21105/joss.01686.

[ejn70624-bib-0040] Widmann, A. , E. Schröger , and N. Wetzel . 2026. “Measuring the Genuine Mismatch Negativity in the Auditory Multi‐Feature Paradigm.” European Journal of Neuroscience 63, no. 1: e70362. 10.1111/ejn.70362. 41492177 PMC12769272

[ejn70624-bib-0041] Willmore, B. D. B. , and A. J. King . 2023. “Adaptation in Auditory Processing.” Physiological Reviews 103, no. 2: 1025–1058. 10.1152/physrev.00011.2022. 36049112 PMC9829473

[ejn70624-bib-0042] Wolff, C. , and E. Schröger . 2001. “Activation of the Auditory Pre‐Attentive Change Detection System by Tone Repetitions With Fast Stimulation Rate.” Cognitive Brain Research 10, no. 3: 323–327. 10.1016/S0926-6410(00)00043-4. 11167055

[ejn70624-bib-0043] Yeark, M. , B. Paton , and J. Todd . 2021. “The Influence of Variability on Mismatch Negativity Amplitude.” Biological Psychology 164: 108161. 10.1016/j.biopsycho.2021.108161. 34333068

